# Hospital admission for type 2 diabetes mellitus under the Universal Coverage Scheme in Thailand: A time- and geographical-trend analysis, 2009–2016

**DOI:** 10.1371/journal.pone.0253434

**Published:** 2021-07-01

**Authors:** Tanapat Laowahutanon, Haruyo Nakamura, Hisateru Tachimori, Shuhei Nomura, Tippawan Liabsuetrakul, Apiradee Lim, Petch Rawdaree, Netnapis Suchonwanich, Hiroyuki Yamamoto, Aya Ishizuka, Kenji Shibuya, Hiroaki Miyata, Virasakdi Chongsuvivatwong

**Affiliations:** 1 National Health Security Office, Bangkok, Thailand; 2 International Development Center of Japan Inc., Tokyo, Japan; 3 Institute for Global Health Policy Research (iGHP), Bureau of International Health Cooperation National Center for Global Health and Medicine, Shinjuku City, Japan; 4 Endowed Course for Health System Innovation, Keio University School of Medicine, Minato City, Japan; 5 Department of Clinical Epidemiology, Translational Medical Center, National Center of Neurology and Psychiatry, Kodaira, Japan; 6 Department of Health Policy and Management, Keio University School of Medicine, Minato City, Japan; 7 Department of Global Health Policy, Graduate School of Medicine, The University of Tokyo, Tokyo, Japan; 8 Epidemiology Unit, Faculty of Medicine, Prince of Songkla University, Hat Yai, Songkhla, Thailand; 9 Department of Mathematics and Computer Science, Faculty of Science and Technology, Prince of Songkla University Pattani Campus, Pattani, Thailand; 10 Department of Internal Medicine, Faculty of Medicine Vajira Hospital, Navamindradhiraj University, Bangkok, Thailand; 11 The Health Intervention and Technology Assessment Program (HITAP), Nonthaburi, Thailand; 12 Department of Healthcare Quality Assessment, Graduate School of Medicine, The University of Tokyo, Bunkyo City, Japan; 13 Soma COVID Vaccination Medical Center, Fukushima, Japan; Istituto di Ricerche Farmacologiche Mario Negri, ITALY

## Abstract

**Background:**

Descriptive analyses of 2009–2016 were performed using the data of the Universal Coverage Scheme (UCS) which covers nearly 70 percent of the Thai population. The analyses described the time and geographical trends of nationwide admission rates of type 2 diabetes mellitus (T2DM) and its complications, including chronic kidney disease (CKD), myocardial infarction, cerebrovascular diseases, retinopathy, cataract, and diabetic foot amputation.

**Methods and findings:**

The database of T2DM patients aged 15–100 years who were admitted between 2009 and 2016 under the UCS and that of the UCS population were retrieved for the analyses. The admitted cases of T2DM were extracted from the database using disease codes of principal and secondary diagnoses defined by the International Classification of Diseases 9th and 10th Revisions. The T2DM admission rates in 2009–2016 were the number of admissions divided by the number of the UCS population. The standardized admission rates (SARs)were further estimated in contrast to the expected number of admissions considering age and sex composition of the UCS population in each region. A linearly increased trend was found in T2DM admission rates from 2009 to 2016. Female admission rates were persistently higher than that of males. In 2016, an increase in the T2DM admission rates was observed among the older ages relative to that in 2009. Although the SARs of T2DM were generally higher in Bangkok and central regions in 2009, except that with CKD and foot amputation which had higher trends in northeastern regions, the geographical inequalities were fairly reduced by 2016.

**Conclusion:**

Admission rates of T2DM and its major complications increased in Thailand from 2009 to 2016. Although the overall geographical inequalities in the SARs of T2DM were reduced in the country, further efforts are required to improve the health system and policies focusing on risk factors and regions to manage the increasing T2DM.

## Introduction

In the world, approximately 451 million people aged 18 to 99 years lived with diabetes in 2017 [1], and this number is projected to further increase to 693 million by 2045 [1]. When diabetes is not properly managed, complications develop typically in kidney failure, vision loss, and foot amputation. Diabetes has increasingly become a global burden of disease that increases premature death, reduces quality of life and drives up healthcare cost [2]. The World Health Organization (WHO) emphasizes importance of prevention and early diagnosis of diabetes, particularly for type 2 diabetes mellitus (T2DM) which can be effectively reduced through population-based and individual prevention measures that target key risk factors [3].

In Thailand, prevalence of diabetes increased from 2.3% in 1991 [4] to 9.6% (6.5 million diabetes cases) in 2016 [5]. More than 94.0% of diabetic cases in the country are T2DM [2] and the six major diabetic complications that are annually screened in the country include chronic kidney disease (CKD), myocardial infarction (MI), cerebrovascular diseases, retinopathy, cataract and foot amputation [6]. The National Health Examination Survey in 2014 found that only 23.5% of people with T2DM were treated with fasting plasma glucose being less than 130 mg/dL, while 43.0% of them were undiagnosed [7].

Thailand has successfully reformed its health service system by equitably redistributed health professionals, health infrastructure development, and rural retention policies over the past four decades [8]. Thailand has achieved remarkable improvements in population health since the almost achievement of universal health coverage (UHC) in 2002. The UHC is a concept that indicates a state in which all citizens are covered by some kind of health insurance. To achieve the UHC, Thailand uses the three major health protection schemes, which are the Civil Service Medical Benefit Scheme (CSMBS), the Social Security Scheme (SSS), and the Universal Coverage Scheme (UCS) [9]. These three major schemes, i.e., the CSMBS, the SSS, and the UCS, cover over 97% of Thai citizens in the year 2016, and the last one, the UCS, taken care of by the National Health Security Office (NHSO), covers more than 48 million people, approximately 70% of the Thai population as of 2016 [9]. The public health insurance status of Thai citizens will be centrally managed by the NHSO, so that Thai citizens must have only one public health insurance, though people have possible to add private health insurance(s) by themselves to a public one. When comparing proportions of the population utilizing UCS or other government (public) health insurance schemes, classified by gender and age group, the findings showed that most of the UCS people were children and elderly (0–19 years old and people over 58 years old) while most of the other schemes were being utilized by the working-aged group 25–49 years old. Government and civil servants’ rights are distributed in all age groups, especially 40 years old [9].

**Fig 1:**
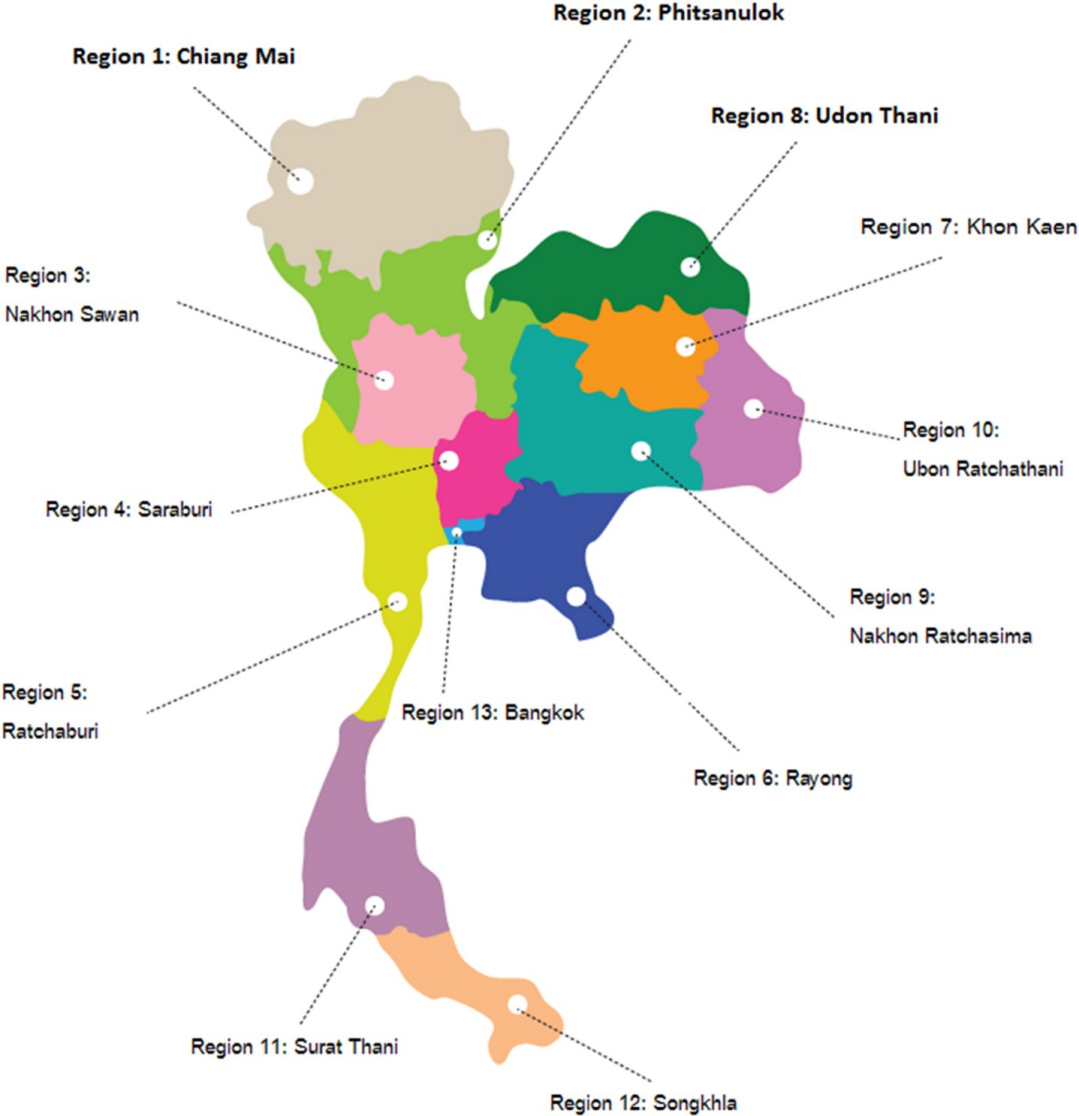
National health security organization regions.

NHSO has 13 Regional Offices (see Fig 1) sharing all resources and regulation [10]. The UCS is financed by general tax revenue. The UCS offers the patients with T2DM and its complications the comprehensive benefit package which includes prevention, curative and rehabilitation services. The beneficiaries are systematically required to visit the registered primary care facility as the first point of contact. In case of severe conditions, they are referred to secondary and tertiary care facilities [11]. The UCS applies mixed-method provider payments, with mainly close-ended capitation for outpatient care and based on diagnosis-related groups, with a global budget, for inpatient care [12].

Despite the national efforts in the implementation of UCS, there is evidence of a rise in intra-national health inequalities in diabetic mortality rates in Thailand [13]. However, the level of health inequalities in morbidity measures such as hospital admission has not been well documented. To fill the knowledge gap, in this paper, we 1) described for the first time a time-trend analysis of nationwide admission rates of T2DM and its six major diabetic complications among the UCS beneficiaries in Thailand from 2009 to 2016, and 2) assessed regional inequality in trend of the admission rates across the 13 NHSO Regions.

## Materials and methods

### Data

The data set stored at the NHSO compiled from three data sources were used for this study. Two are reports sent from hospitals to the Ministry of Public Health, namely the hospital admission database and the operation database, which include personal national identification number, sex, date of birth, the NHSO Region, province of hospitalization, hospital code of registration, hospital code of admission, date of admission, date of discharge, date of death, and principal and secondary diagnoses. The remaining is compiled from hospital reports sent to the NHSO for reimbursement. All data were fully anonymized before we accessed them.

All Thai citizens entitled to use the UCS are registered in a special table of the NHSO datasets. These are updated annually against birth and death registries taken care by the Ministry of Interior. Although the NHSO database contains admissions of patients covered by all medical benefit schemes, only those entitled to the UCS were used for this study. These data are regularly checked to prevent duplication based on personal national ID number, names and birthdates. After careful data cleaning, a total of 4,297,321 T2DM admitted cases of 2,689,642 UCS patients aged 15 to 100 years in Thailand between 2009 and 2016 were included in this study. Although Thailand achieved UHC in 2002, we decided to use only data from 2009 onwards for our analyses since there were a number of missing values and errors in data before 2009 [14, 15]. Ages of below 15 years were excluded because T2DM was rare in children until recently [16] and above 100 years were considered to be primarily caused by typing errors. All subsequent analyses were done on data of the UCS population as denominators and those of the UCS admissions for T2DM as numerators. The UCS population of 15 to 100 years of age represents approximately 54.0% of the total population of 2009–2016 in Thailand.

### Definitions of T2DM and its complications

A trained medical statistic officer at the hospitals entered diagnosis of T2DM and its complications, CKD, MI, cerebrovascular diseases, retinopathy and cataract based on the International Classification of Diseases 10th Revision (ICD-10) [17], and diabetic foot amputation based on the International Classification of Diseases 9th Revision (ICD-9) CM vol. 3 Procedure Codes [18]. All UCS admitted cases whose principal or secondary diagnosis was coded as T2DM (E11.1 to E11.9), with or without CKD (N18.1 to N18.6, N18.9, E11.2, E14.2, N08.3, N19 and N18.9), MI (I21 and I22), cerebrovascular diseases (I60 to I69), retinopathy (H36.0), cataract (H25.0 to H25.2, H25.8, H25.9, H26.0 to H26.4, H26.8, H26.9, and H28.0), or diabetic foot amputations (84.10–84.17) were included, and any other cases were excluded from this study. Stage 3 or higher stages of CKD are usually considered as diabetic complications. However, stage 1 and 2 of CKD were also included in this study because the ICD-10 code, E11.2 includes all stages of kidney complications, and thus it was impossible to exclude stage 1 and 2 of CKD cases. T2DM cases with acute, as well as subsequent MI were included in this study. While only H36.0, diabetic retinopathy was considered as a diabetic complication, all types of cataract were included in this study because diabetic cataract is often misdiagnosed as other types of cataract. Diagnosis of foot amputation was considered as a diabetic complication if it was performed from the toe to above the knee.

### Data analysis

Descriptive analyses using the retrospective data were performed to summarize age, sex and regional structure of the UCS patients who were admitted for T2DM between 2009 and 2016 in Thailand and the trends in 2009 and 2016 were compared to depict the change in the trends over the eight years. To check the existence of a linear temporal trend, we also conducted a regression analysis with the number of people as the dependent variable and time (year) as the explanatory variable, and showed the regression coefficients β_t_ and their p-values to Table 1.

**Table 1:** Number and demographic characteristics of the Universal Coverage Scheme patients admitted for type 2 diabetes mellitus in 2009–2016.

	2009	2010	2011	2012	2013	2014	2015	2016	Regression analysis^1^	
	n (%) or mean (SD)	n (%) or mean (SD)	n (%) or mean (SD)	n (%) or mean (SD)	n (%) or mean (SD)	n (%) or mean (SD)	n (%) or mean (SD)	n (%) or mean (SD)	β_t_	p
Sex										
Female	181,402	193,297	202,297	208,632	219,830	228,533	239,283	247,671	9330	p < 0.0001
	(66.1)	(65.8)	(64.9)	(64.2)	(63.6)	(63.1)	(62.9)	(62.4)		
Male	92,938	100,420	109,310	116,190	125,870	133,426	141,304	149,239	8102	p < 0.0001
	(33.9)	(34.2)	(35.1)	(35.8)	(36.4)	(36.9)	(37.1)	(37.6)		
Age										
15–19	364	355	393	373	393	449	489	478	19.7	0.0014
	(0.1)	(0.1)	(0.1)	(0.1)	(0.1)	(0.1)	(0.1)	(0.1)		
20–24	547	608	628	703	755	776	801	876	44.8	p < 0.0001
	(0.2)	(0.2)	(0.2)	(0.2)	(0.2)	(0.2)	(0.2)	(0.2)		
25–29	1,113	1,104	1,133	1,209	1,253	1,335	1,486	1,551	67.0	p < 0.0001
	(0.4)	(0.4)	(0.4)	(0.4)	(0.4)	(0.4)	(0.4)	(0.4)		
30–34	2,478	2,582	2,632	2,696	2,888	2,913	3,126	3,156	101	p < 0.0001
	(0.9)	(0.9)	(0.8)	(0.8)	(0.8)	(0.8)	(0.8)	(0.8)		
35–39	5,603	5,938	5,976	6,100	6,233	6,541	6,739	6,822	171	p < 0.0001
	(2.0)	(2.0)	(1.9)	(1.9)	(1.8)	(1.8)	(1.8)	(1.7)		
40–44	11,867	12,206	12,351	12,733	13,053	13,424	13,980	13,787	308	p < 0.0001
	(4.3)	(4.2)	(4.0)	(3.9)	(3.8)	(3.7)	(3.7)	(3.5)		
45–49	20,490	21,236	21,809	22,798	23,893	24,565	25,236	25,353	755	p < 0.0001
	(7.5)	(7.2)	(7.0)	(7.0)	(6.9)	(6.8)	(6.6)	(6.4)		
50–54	30,357	31,468	32,063	33,243	35,080	36,049	37,860	39,043	1269	p < 0.0001
	(11.1)	(10.7)	(10.3)	(10.2)	(10.1)	(10.0)	(9.9)	(9.8)		
55–59	39,833	42,140	43,507	44,951	46,174	47,384	49,119	50,468	1455	p < 0.0001
	(14.5)	(14.3)	(14.0)	(13.8)	(13.4)	(13.1)	(12.9)	(12.7)		
60–64	42,231	46,458	50,187	53,045	56,747	59,059	61,020	63,304	2984	p < 0.0001
	(15.4)	(15.8)	(16.1)	(16.3)	(16.4)	(16.3)	(16.0)	(15.9)		
65–69	41,038	43,357	45,925	47,328	51,427	55,209	60,056	64,124	3298	p < 0.0001
	(15.0)	(14.8)	(14.7)	(14.6)	(14.9)	(15.3)	(15.8)	(16.2)		
70–74	37,359	40,155	42,922	44,155	46,068	47,329	48,787	50,920	1824	p < 0.0001
	(13.6)	(13.7)	(13.8)	(13.6)	(13.3)	(13.1)	(12.8)	(12.8)		
75–79	24,996	27,241	30,523	32,103	34,926	37,129	39,243	40,946	2313	p < 0.0001
	(9.1)	(9.3)	(9.8)	(9.9)	(10.1)	(10.3)	(10.3)	(10.3)		
80–84	11,356	13,362	15,093	16,484	18,459	20,367	21,998	24,058	1784	p < 0.0001
	(4.1)	(4.5)	(4.8)	(5.1)	(5.3)	(5.6)	(5.8)	(6.1)		
85+	4,708	5,507	6,465	6,901	8,351	9,430	10,647	12,024	1039	p < 0.0001
	(1.7)	(1.9)	(2.1)	(2.1)	(2.4)	(2.6)	(2.8)	(3.0)		
Mean (SD)	62.4	62.6	63	63.1	63.3	63.5	63.7	63.9	-	-
	(12.0)	(12.1)	(12.1)	(12.1)	(12.2)	(12.2)	(12.3)	(12.3)		
NHSO Regions										
1. Chiang Mai	19,572	21,232	22,492	23,021	23,995	25,160	26,715	28,366	1166	p < 0.0001
	(7.1)	(7.2)	(7.2)	(7.1)	(6.9)	(7.0)	(7.0)	(7.1)		
2. Phitsanulok	14,631	15,857	16,760	18,195	19,024	19,455	20,411	20,937	903	p < 0.0001
	(5.3)	(5.4)	(5.4)	(5.6)	(5.5)	(5.4)	(5.4)	(5.3)		
3. Nakhon Sawan	13,592	15,699	16,764	17,646	17,999	18,288	18,974	19,865	776	0.0002
	(5.0)	(5.3)	(5.4)	(5.4)	(5.2)	(5.1)	(5.0)	(5.0)		
4.Saraburi	23,401	25,965	26,257	25,344	26,249	27,420	28,995	30,772	847	0.0014
	(8.5)	(8.8)	(8.4)	(7.8)	(7.6)	(7.6)	(7.6)	(7.8)		
5. Ratchaburi	27,208	28,203	30,047	29,901	31,538	32,479	33,122	34,033	968	p < 0.0001
	(9.9)	(9.6)	(9.6)	(9.2)	(9.1)	(9.0)	(8.7)	(8.6)		
6. Rayong	22,439	24,207	26,634	27,231	28,588	29,743	30,891	33,143	1417	p < 0.0001
	(8.2)	(8.2)	(8.5)	(8.4)	(8.3)	(8.2)	(8.1)	(8.4)		
7. Khon Kaen	27,623	30,320	32,205	34,357	36,748	39,207	40,608	41,217	2024	p < 0.0001
	(10.1)	(10.3)	(10.3)	(10.6)	(10.6)	(10.8)	(10.7)	(10.4)		
8. Udon Thani	27,032	28,833	30,200	30,522	33,339	35,563	37,644	38,258	1685	p < 0.0001
	(9.9)	(9.8)	(9.7)	(9.4)	(9.6)	(9.8)	(9.9)	(9.6)		
9. Nakhon Ratchasima	26,587	27,945	30,292	33,091	36,295	38,139	41,198	44,157	2571	p < 0.0001
	(9.7)	(9.5)	(9.7)	(10.2)	(10.5)	(10.5)	(10.8)	(11.1)		
10. Ubon Ratchathani	20,241	20,368	22,361	23,425	25,384	26,835	29,299	30,886	1602	p < 0.0001
	(7.4)	(6.9)	(7.2)	(7.2)	(7.3)	(7.4)	(7.7)	(7.8)		
11. Surat Thani	14,498	15,659	16,723	18,423	19,504	21,075	21,777	22,817	1226	p < 0.0001
	(5.3)	(5.3)	(5.4)	(5.7)	(5.6)	(5.8)	(5.7)	(5.7)		
12. Songkhla	14,515	14,903	15,888	17,570	19,069	19,613	20,591	21,288	1054	p < 0.0001
	(5.3)	(5.1)	(5.1)	(5.4)	(5.5)	(5.4)	(5.4)	(5.4)		
13. Bangkok	23,001	24,526	24,984	26,096	27,968	28,982	30,362	31,171	1193	p < 0.0001
	(8.4)	(8.4)	(8.0)	(8.0)	(8.1)	(8.0)	(8.0)	(7.9)		
Total	274,340	293,717	311,607	324,822	345,700	361,959	380,587	396,910	17432	p < 0.0001

^1^ Regression analysis with the number of people as the dependent variable and year as the explanatory variable were conducted to check the existence of a linear temporal trend. The *β_t_* is the slope parameter of the variable that represents the year.

Note: The Universal Coverage Scheme (UCS) patients admitted for T2DM are the UCS beneficiaries who were admitted for type 2 diabetes mellitus (T2DM) between 2009 and 2016. The number of UCS patients was counted as one in a year. That is, a UCS patient who was admitted for T2DM for multiple times in a year was counted as one in that year. If the same patient was admitted for T2DM in another year, he/she was counted as one again in the separate year.

We estimated the standardized admission rate (SAR) of each region using the following equation for each region.

$${SAR}_{i}=\frac{o_{i}}{e_{i}}$$

$$e_{i}=\sum_{j=1}^{J}p_{j}n_{ij}$$

where *SAR*_*i*_ is standardized admission rate in region *i*; *o*_*i*_ is the observed number of admissions in region *i*; *e*_*i*_ is the expected number of admissions in region *i*; *j* is the population stratum defined by age and sex; *p*_*j*_ is standard admission rate in the 2009 UCS population for the population stratum *j*. Age was categorized into 15 groups in intervals of five years, except the last category that includes 85 to 100 years of age.

To estimate temporal trend of admissions, we conducted time series regressions with the following negative binomial regression model.

$$n_{i}∼NegativeBinomial(\mu_{i},\phi )$$

$$\mu_{i}=exp(\beta_{0}+\beta_{1}x+\log N_{i})$$

where *n_i_* is the number of admissions of *i*th time point, *N_i_* is the number of UC population of *i*th time point, *x* is the indicator variable of time points, μ is the mean parameter and *ϕ* is dispersion parameter of Negative Binomial distribution, *β*_0_ is the intercept, and *β*_1_ is the slope parameter.

R version 3.4.1 (R Foundation for Statistical Computing, Vienna, Austria) [19] and laterwas used to analyze the data. To draw choropleth maps, we used R version 3.4.1 and later with package ‘sf’ [20] and package ‘ggplot2’ [21]. Ethics of the study was approved by the Institutional Review Board of the National Center for Global Health and Medicine (NCGM) in Japan on 11 May 2018 (NCGM-G-002524-00).

## Results

Table 1 presents the number and demographic characteristics of the UCS patients admitted for T2DM in 2009–2016. Over 60% of T2DM patients were female throughout the period, although the sex disparity narrowed in the eight years. The mean age (SD) of the UCS T2DM patients was 63.1 (12.2) years throughout the years and it annually rose by 0.2 years on average. The overall number of UCS patients with T2DM annually increased by 5.4%, and 14.4% among the 85 year-olds and older from 2009 to 2016. The number of T2DM patients proportionately increased in Region 9 (Nakhon Ratchasima), 10 (Ubon Ratchathani) and 11 (Songkhla), and decreased in Region 4 (Saraburi), 5 (Ratchaburi) and 13 (Bangkok) in the study period.

**Fig 2:**
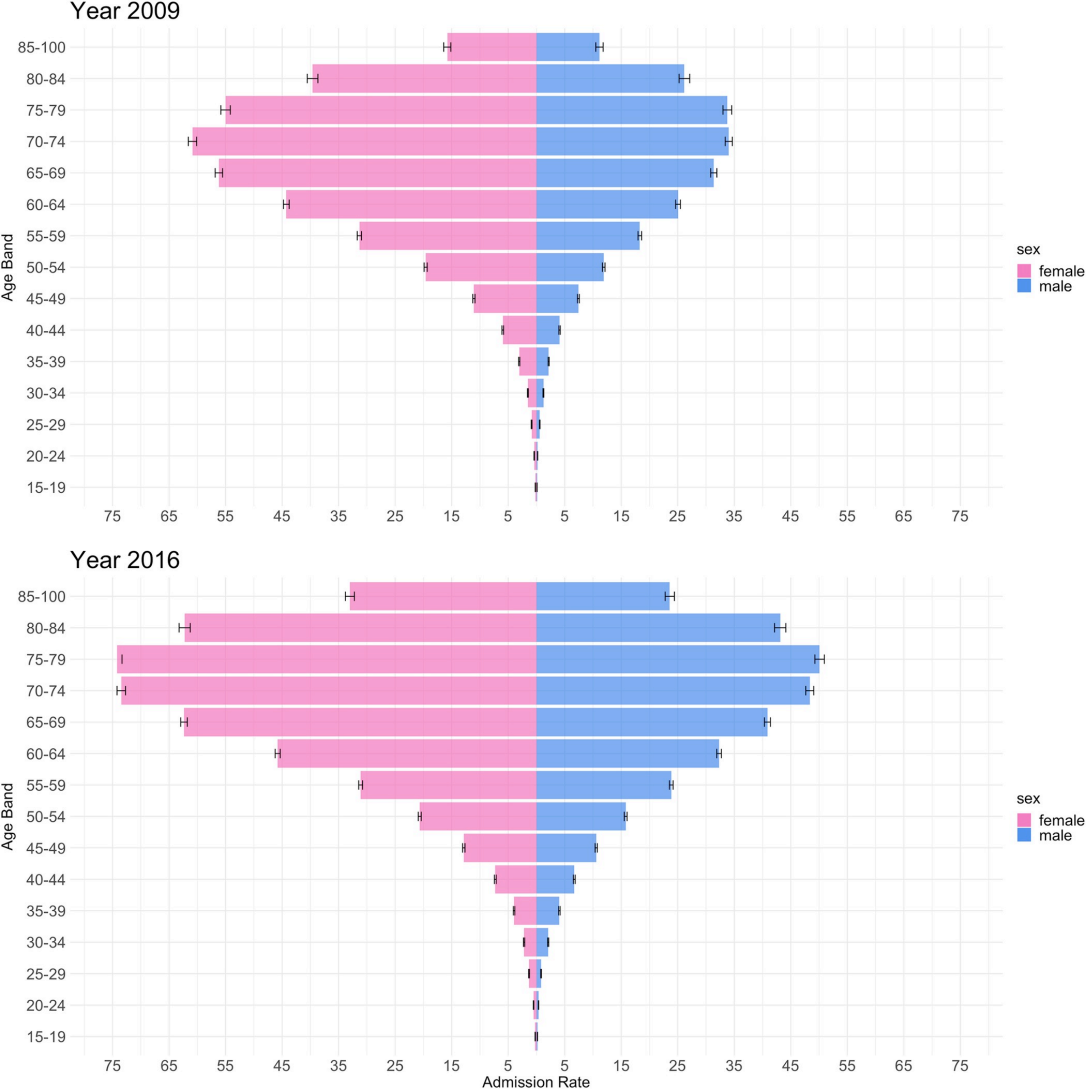
Population pyramids of admitted cases with Type 2 Diabetes Mellitus (T2DM) among the Universal Coverage Scheme patients in Thailand in 2009 and 2016. Note: T2DM crude admission rates are per 1,000 population. The error bars indicate the 99% confidence interval.

Fig 2 shows the population pyramids of admitted cases with T2DM among the UCS beneficiaries in Thailand in 2009 and 2016. The female crude admission rates were persistently higher than that of males both in 2009 and 2016. The sex disparity in the crude admission rates widened between their 30s and 70s. In 2009, the crude admission rates of both sexes started increasing in their late 30s, females reached a peak at the ages of 70 to 74 and males at the ages of 75 to 79, and then both declined. In 2016, both sexes reached a peak at the ages of 75–79. Although the trend was similar in the two years, the crude admission rates were persistently much higher and the overall increase occurred in older ages in 2016.

**Fig 3:**
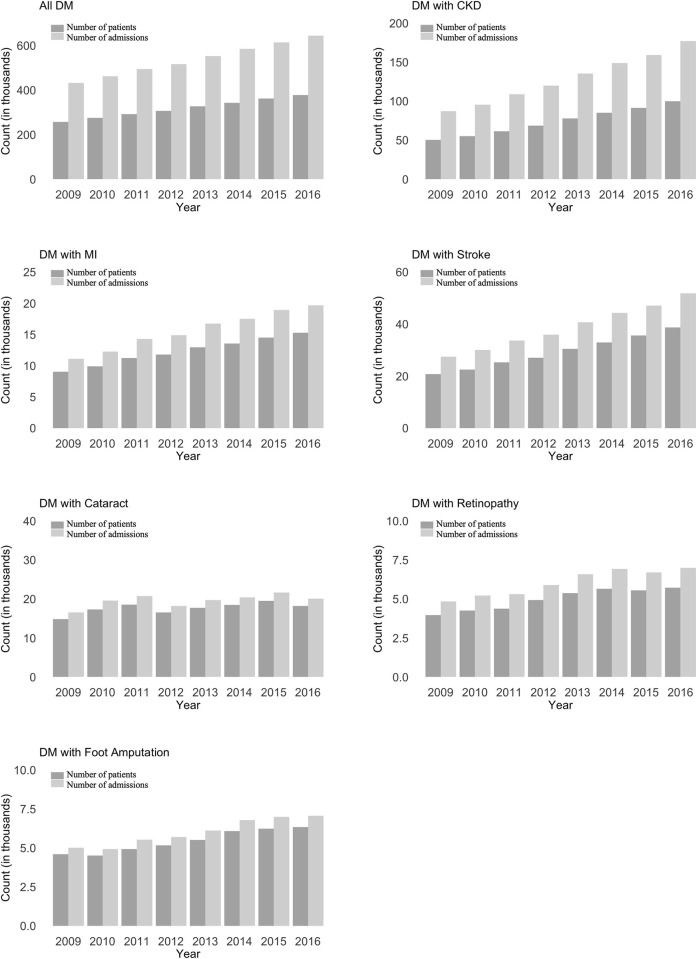
The number of patients and the number of admissions of type 2 diabetes mellitus with the six major complications under the Universal Coverage Scheme in Thailand from 2009 to 2016. Note: The number of admissions refers to how many times the Universal Coverage Scheme (UCS) patients were admitted for type-2 diabetes mellitus (T2DM) with complications in each year, whereas the number of patients refers to how many T2DM patients were admitted in the year. A patient could be admitted for multiple times in a year. The scale for Y-axis varies between panels.

**Fig 4:**
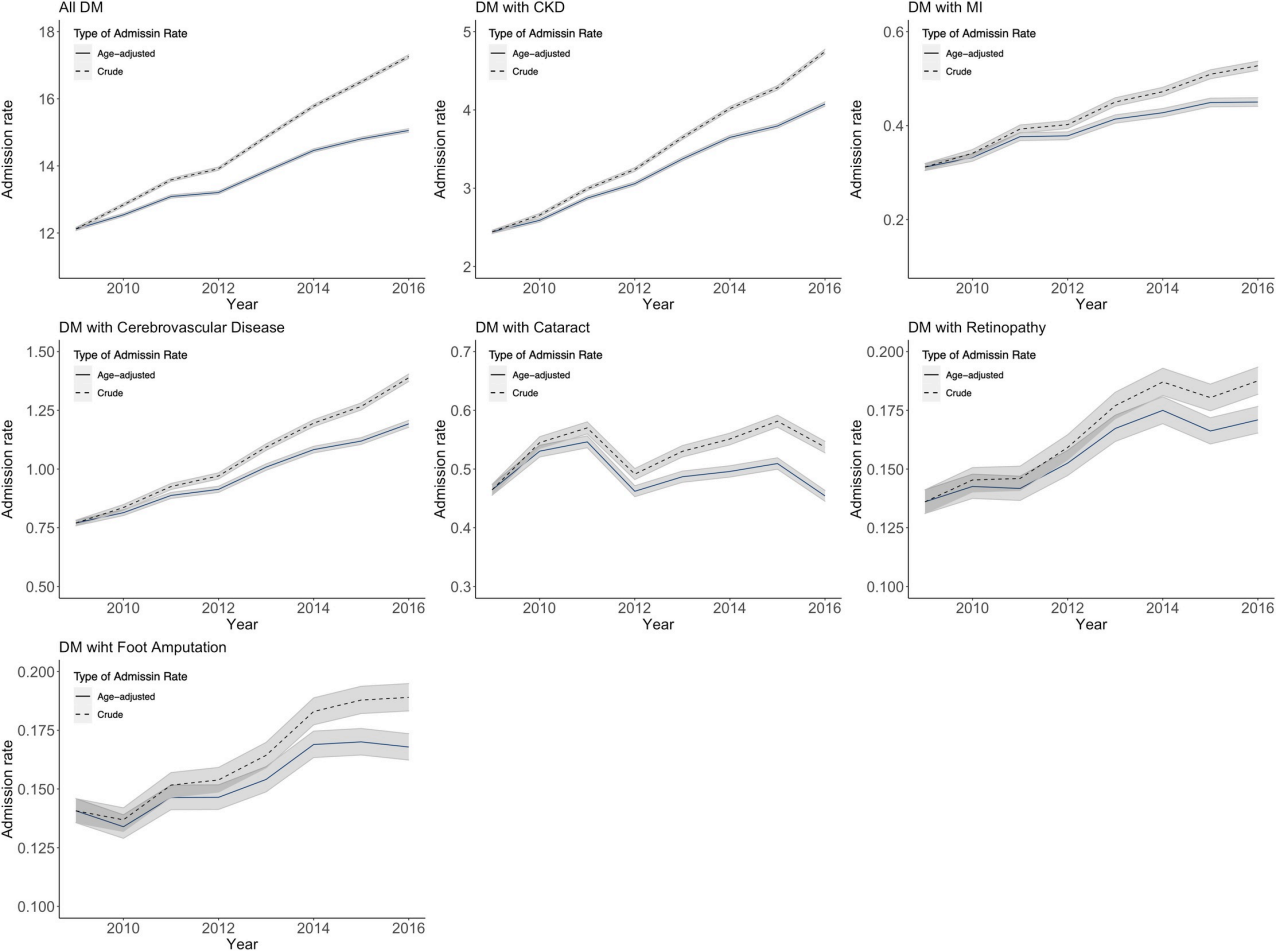
The crude and age-adjusted admission rates of type 2 diabetes mellitus with the six major complications under the Universal Coverage Scheme in Thailand from 2009 to 2016. Note: The crude admission rate is the number of admissions divided by the number of the UCS population. The age-adjusted admission rate is calculated by the direct method with the 2009 population as the standard population. The gray bands indicate the 99% confidence interval.

Figs 3 and 4 presents the number of patients, the number of admissions and the admission rates of T2DM and those with the six major complications: CKD, MI, cerebrovascular diseases, retinopathy, cataract and foot amputation from 2009 to 2016 in Thailand. Overall, there was a linearly increased trend in T2DM crude admission rates by 5.2% annually. Among the T2DM admissions, CKD was present in 24.0% in 2009–2016, and the T2DM crude admission rates with CKD also had a positive linear trend by 10.0% per year. From 2009 to 2016, the average crude admission rates of T2DM with CKD were 3.5/1000, cerebrovascular diseases 1.1/1000, cataract 0.5/1000, MI 0.4/1000, and retinopathy and foot amputation 0.2/1000 population, respectively.

Table 2 presents the results of the time series regressions to estimate temporal trend of admissions showed that all types of T2DM admissions except that with cataract had a significant and positive temporal trend (see also the S1 Table, which shown statistically more detailed results).

**Table 2:** Temporal trend analysis for the number of each admission by year.

Dependent variables	*β*^1^	SE	p value
**All DM admissions**	0.050566	0.001187	p < 0.001
**DM with CKD admissions**	0.095675	0.001861	p < 0.001
**DM with MI admissions**	0.07541	0.00424	p < 0.001
**DM with cerebrovascular diseases admissions**	0.084358	0.001941	p < 0.001
**DM with cataract admissions**	0.015244	0.009283	0.101
**DM with retinopathy admissions**	0.049766	0.005599	p < 0.001
**DM with amputation admissions**	0.050946	0.004584	p < 0.001

^1^ The negative binomial regression analysis with the number of admissions as the dependent variable and year as the explanatory variable was conducted to check the existence of temporal trend of admissions. The β is the slope parameter of the variable that represents the year.

Note: SE: Standard error, DM: Diabetes mellitus, CKD: Chronic kidney disease, MI: Myocardial infarction

Fig 4 also presents the age-adjusted admission rates of T2DM and those with the six major complications. Though the age-adjusted rates were significantly lower than the crude rates of T2DM and those with the six major complications in 2016, the age-adjusted rates in 2016 were significantly higher than those in 2009 except for T2DM with cataract admission. These temporal trends were consistent with those of the crude rates. Table 3 shows the relative change of the age-adjusted admission rate between the year 2009 and 2016. T2DM with CKD admissions had the highest relative change (0.67) followed by T2DM with cerebrovascular disease admissions (0.55) and T2DM with MI admissions (0.45). Those of T2DM, T2DM with retinopathy, and T2DM with foot amputation admissions were around 0.21 to 0.24. On the other hand, the relative change of T2DM with cataract admissions were nearly 0 (-0.02).

**Table 3:** Age-adjusted admission rate, actual change, and relative change between the year 2009 and 2016.

	Age-adjusted admission rate^1^ [99% CI]				Actual	Relative
	2009		2016		change	change
All DM	12.12	[12.07–12.16]	15.06	[15.00–15.11]	2.94	0.24
DM with CKD	2.44	[2.42–2.46]	4.08	[4.05–4.10]	1.64	0.67
DM with MI	0.31	[0.30–0.32]	0.45	[0.44–0.46]	0.14	0.45
DM with Cerebrovascular disease	0.77	[0.76–0.78]	1.19	[1.18–1.21]	0.42	0.55
DM with Cataract	0.46	[0.46–0.47]	0.45	[0.45–0.46]	-0.01	-0.02
DM with Retinopathy	0.14	[0.13–0.14]	0.17	[0.17–0.18]	0.03	0.21
DM with Foot Amputation	0.14	[0.14–0.15]	0.17	[0.16–0.17]	0.03	0.21

^1^ The admission rates are per 1,000 population.

Note: CI: Confidence interval, DM: Diabetes mellitus, CKD: Chronic kidney disease, MI: Myocardial infarction. Actual change was obtained by subtracting 2009 from 2016; relative change was obtained by dividing the actual change by the 2009 value.

**Fig 5:**
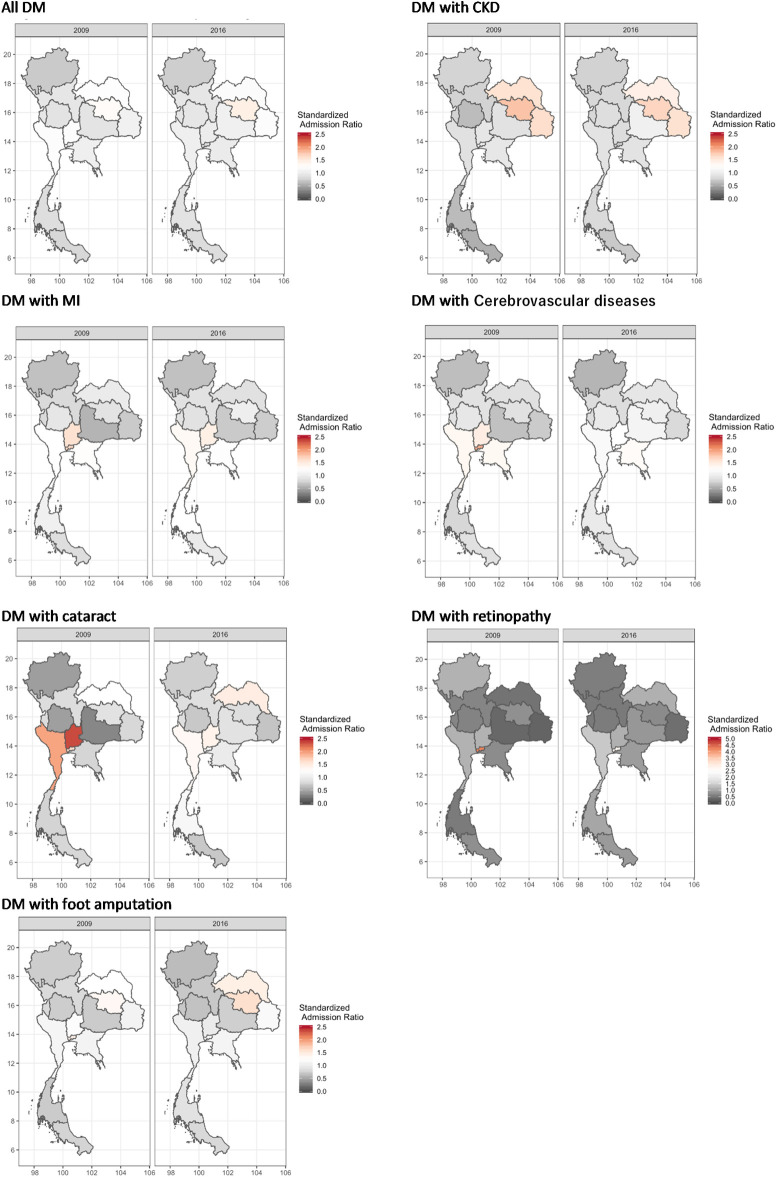
Comparison of age- and sex- standardized admission rate of type 2 diabetes mellitus and its complications in the NHSO Regions in 2009 and 2016. Note: The standardized admission rate (SAR) of type 2 diabetes mellitus (T2DM) and its complications were shown in white if it is the national average. The color changes into red if SAR is higher than the national average, and gray if it is lower than the national average. The scale for SARs of T2DM with retinopathy is different from others: it continues up to 5.0 because SAR of T2DM with retinopathy in Bangkok was substantially higher than other regions in 2009.

Fig 5 presents SARs of T2DM and its complications in the NHSO 13 Regions in 2009 and 2016. The SARs of T2DM showed a regional disparity for both 2009 and 2016, with higher SARs in central and northeastern regions in general. For instance, SARs of T2DM with CKD were higher in northeastern regions (Region 7: Khon Kaen(1.79), Region 8: Udon Thani (1.58), and Region 10: Ubon Ratchathani (1.64)) than other regions of the nation that had rates less than 1.00 in 2009. In addition, the SARs of T2DM with cataract were high in Bangkok (Region 13) (1.64 in 2009, 1.25 in 2016) and other central regions (Region 4 (2.35 in 2009, 1.40 in 2016) and Region 5 (1.95 in 2009, 1.33 in 2016)). Furthermore, The SAR of T2DM with retinopathy in Bangkok (Region 13) was 4 times higher than the national average in 2009.

In general, all regions showed a decline in SARs of T2DM in the period between 2009 to 2016, except for Region 7 (Khon Kaen) that showed an increase from 1.22 to 1.44 and Region 9 (Nakhon Ratchasima) that increased from 0.88 to 1.05. As for the SARs for the complications of T2DM, most regions showed a decreasing trend for each of the complications except for SAR of T2DM with CKD in Region 9 that increased from 0.86 to 1.05 in the same period; MI in Region 7 that increased from 0.88 in 2009 to 1.01 in 2016; cerebrovascular diseases that increased in Regions 7 and 9 between 2009 and 2016, from 0.83 to 1.03 and 0.65 to 1.11, respectively; retinopathy that increased in Regions 8 and 11 (Surat Thani) from 0.43 to 1.11 and 0.52 to 1.00, respectively; foot amputation that in northeastern regions of Regions 7 and 8 that increased from 1.35 to 1.61 and 1.24 to 1.43, respectively.

## Discussion

A linearly increased trend of T2DM admission rates and that with the six major diabetic complications were found from 2009 to 2016 in Thailand. Female admission rates were persistently higher than that of males. In 2016, overall increase in the T2DM admission rates was observed among the older ages relative to that in 2009. Although geographical inequalities in the T2DM admission rates were found, the reduced trend in the inequalities was also observed between 2009 and 2016.

The observed sex disparities in frequency of the T2DM admissions were consistent with the National Health Examination Surveys [2] and an assessment on quality of care among patients diagnosed with T2DM and hypertension, which presented that females in Bangkok were 1.13 times more likely to have HbA1C level of higher than 9.0% [22]. Biology might play a part in observed sex disparities as females typically transition from prediabetes to diabetes with a worse cardiovascular risk profile and a higher BMI than males. However, psychosocial factors, such as health-seeking behavior and provision of health care, play more important part in the differences, which can be addressed through changes in policy and health-care delivery [23]. It should be noted that high admission rate does not necessarily mean high prevalence of the disease, as previous studies showed higher percentage of undiagnosed diabetes [2] and slightly higher fasting plasma glucose among males in Thailand [24].

While the number of patients, the number of admissions and admission rates of T2DM steadily increased from 2009 to 2016, the increased trend in the sex-and age-adjusted T2DM admission rates, which were estimated using the national UCS population of 2009 as the standard population, (12.1 in 2009 and 15.0 in 2016, *results not shown*) were rather gradual as compared with the numbers of crude admissions rates (12.1 in 2009 and 17.3 in 2016). This result suggests that the increase in the T2DM admission rates is partly due to the increased and aged population of the country. Although further studies are required, it could imply that Thailand may face the greater burden of T2DM in the future if the trend of population growth and aging continues in the country.

The T2DM admission rates reached a peak at the ages of 70s and then declined in 2009 and 2016 presumably due to premature death of the T2DM patients. The shifted trend of the peak age toward elderly among females between 2009 and 2016 can be explained by the fact that the T2DM patients had aged and their longevity had been extended over the eight years [25]. This trend further implies the need of increased costs of providing diabetes-related care as older adults with diabetes is clearly more complicated with multiple coexisting medical conditions, particularly macrovascular complications such as acute myocardial infarction and cerebrovascular diseases and end-stage renal disease [26]. Age also affects the potential risks of overtreatment of hyperglycemia in the hospital, which often leads to longer hospitalization, higher medical costs and increased mortality [27, 28].

Among the six diabetic complications, CKD showed the most significant increase between 2009 and 2016. This trend is partly attributed to the fact that renal replacement therapy including renal and peritoneal dialysis and kidney transplantation required the UCS patients with a co-payment until 2008 [29], which may have inhibited some patients from accessing to proper care until then. In fact, additional 2.5 billion baht (US$ 76 million) was allocated to renal-replacement therapy with 8000 patients receiving haemodialysis and 4000 receiving peritoneal dialysis, after the renal-replacement therapy was included in the scheme [29]. Although Thailand has launched the “Thailand Healthy Lifestyle Strategy 2011–2020 Plan” [30] to reduce the prevalence, complications, disability, mortality and cost of non-communicable diseases including diabetes, it took some time for the national screening and prevention program to be undertaken in the country [4]. Thailand should take this trend seriously as this type of complication is associated with a substantial burden in terms of mortality, morbidity and healthcare cost as it often requires costly and long-term care including dialysis. To prevent progression of CKD stage, the country should strengthen an effective measure, such as glycated hemoglobin control (HbA1c) ≦7.0% [22], as instructed in the Clinical Practice Recommendation for the Evaluation and Management of Chronic Kidney Disease in Adults 2015 [31]. This study also found that the number of admitted cases with CKD were 1.7 times greater than the number of patients. This indicates that many of the patients with diabetic complication of CDK were readmitted, and implies that there might be unmet needs of inpatient care for the T2DM patients with CKD. Further study should be conducted to investigate the reasons behind the frequent readmissions and take measures to meet the needs of the patients.

While the SARs of T2DM were higher in Bangkok and central regions relative to other regions in 2009, except those with CKD and partly foot amputation, they declined in most of the regions by 2016. Additionally, there was an overall trend of SAR reduction in Bangkok and central regions, where human and financial resources were traditionally concentrated, and increase in northeastern regions, where the resources were traditionally scarce, over the eight years [10, 32]. This trend indicates Thailand’s successful health reform by reducing geographical inequalities in inpatient care, which might be a result of equitably redistributed health professionals, health infrastructure development and rural retention policies over the past four decades [8].

On the other hand, the persistently high SAR of T2DM with retinopathy in Bangkok is presumably due to high density of specialists as half of 1,500 ophthalmologists, including 200 retinal specialists, practice in Bangkok [33].

The persistently high SAR of T2DM with CKD in northeastern regions was consistent with a previous study and partly attributed to high prevalence of CKD in northeastern regions (10.8%) relative to other regions (north 8.9%, south 8.1% and Bangkok 6.2%) [34], but partly to an association with lower density of physicians and rurality of the region [14]. The density of physicians in northeastern regions is the lowest in the country [8], as low as seven times lower than Bangkok [10]. It is reasonable to assume that in a region where physicians are scarce, T2DM patients with CKD are unlikely to receive timely, thorough and effective treatment, and consequently deteriorate in conditions. This assumption might explain the high readmission rates of T2DM with CDK, and the highest mortality rates due to diabetes in northeastern regions as found in another study [13]. Moreover, rurality of the northeastern regions, where 71.0% of the population reside in rural setting (north 65.6%, south 66.5% and Central 54.5%) [35], might have halted them from accessing adequate primary care. A previous study found a strong association between the high SAR of diabetes and rurality as the rural population tends to have lack of public transport alternatives and poor health literacy with less education which often limit accessibility to health care. The study also suggested that the percentage of patients who had received up to secondary education was lower in rural districts by approximately 10% [14].

The services covered by the UCS have been expanded, albeit with limitations compared to other schemes, to include not only the basic benefit package but also the special benefits package for specific groups were developed such as HIV/AIDS, high cost of treatment groups, for example, heart surgery, cataract surgery including access to necessary and high-cost medicines [36]. The difference of covered health care services may impede access to health care. This may have the following effects on the results of this study.

1) Withholding medical attention until the last minute may lead to lower estimated admission prevalence rates than in other health care schemes.

2) Complication incident rates might be higher than for those in other health schemes, due to restraint in the use of medical services until a more severe illness occurs. This may make the admission prevalence in the UCS population increase.

Inaccessibility to the outpatient data and absence of information indicating direct causality between diabetes and complicated conditions were the major limitations of this study. For the first limitation, it is important to monitor the long-term trends of diabetic morbidity starting at onset of disease, accessibility and quality of outpatient and inpatient care, and health outcome including mortality to assess the quality of T2DM healthcare in the country. Besides, community involvement in diabetic care should be also carefully monitored, as approximately 77.0% of cost is involved in non-medical activities [37], and community-based screening, study and health promotion would be increasingly important for diabetic care [4]. According to the National Health Examination Survey, conducted from 2004 to 2014 in Thailand, age-adjusted prevalence of diabetes increased from 7.7% in 2004 to 7.8% in 2009 and 9.9% in 2014 (8.9% among men and 10.8% among women) [38]. The number of Thai patients with diabetes is predicted to increase from 1.5 million in 2000 to 2.7 million in 2030 [39]. In this study, we looked at the trend of the inpatient care and tried to capture that of a series of healthcare, but future study should carefully assess the situations of accessibility and quality of T2DM outpatient care, and coordination of outpatient and inpatient care for the most cost-effective T2DM healthcare policies in Thailand.

For the second limitation, we regarded the complications as if they were directly caused by T2DM, when we found T2DM as either principle or secondary diagnosis and one or more of the major complications in an individual record of the hospital admission database since it was the only available information. The data used in this study confirm that each complication is comorbid with diabetes, but do not allow us to determine whether it is caused by diabetes. For example, if a foot amputation was performed, it is not possible to distinguish from the data whether the amputation was due to diabetic necrosis or to something else in a person with diabetes. Therefore, although the ICD-10 codes used in this study to identify comorbidities were carefully designed to exclude as much as possible those with a low association with diabetes, the prevalence of complications may still be an overestimate. Particular attention should be paid to this possibility, especially in the case of amputation.

For the third limitation, we mention the well-known limitation of administrative databases to underestimate the complications rate due to not always perfect ICD coding. Given the above three limitations, the complications reported here may have been overestimated or underestimated.

Thailand has achieved great improvement in health care reform invested on equitable health finance and increased total budget for health expenditures in T2DM and its complications. It is time for the country to carefully identify the risk factors and regions in particular needs of care for T2DM and its complications, and plan on the effective and efficient health care which would not leave no one behind in the country. The results of this study reviled admission rates of T2DM and its major complications increased among the USC population in Thailand from 2009 to 2016 and the overall geographical inequalities in the SARs of T2DM were reduced in the country but remains. These results would be the basis for planning and acting in terms of necessary health provision and preventive measures.

## Supporting information

S1 Table Time series regressions parameters of temporal trend analysis for admissions. (DOCX)
