# Supplementary material for: Hospital admission for type 2 diabetes mellitus under the Universal Coverage Scheme in Thailand: A time- and geographical-trend analysis, 2009–2016
Source: PLoS One. 2021 Jul 1;16(7):e0253434. doi: 10.1371/journal.pone.0253434 (PMC8248737; doi:10.1371/journal.pone.0253434)
Supplement: S1 Table — (DOCX) [file pone.0253434.s001.docx]

**S1 Table . Time series regressions parameters of temporal trend analysis for admissions**

| Parameters^1^ | Estimate | SE | z value | p value |
| --- | --- | --- | --- | --- |
| All DM admissions | | | | |
| $\boldsymbol{\beta}_{\boldsymbol{0}}$: intercept | -4.460529 | 0.006002 | -743.16 | p < 0.001 |
| $\boldsymbol{\beta}_{\boldsymbol{1}}$: time point | 0.050566 | 0.001187 | 42.61 | p < 0.001 |
| DM with CKD admissions | | | | |
| $\boldsymbol{\beta}_{\boldsymbol{0}}$: intercept | -6.108389 | 0.009446 | -646.64 | p < 0.001 |
| $\boldsymbol{\beta}_{\boldsymbol{1}}$: time point | 0.095675 | 0.001861 | 51.41 | p < 0.001 |
| DM with MI admissions | | | | |
| $\boldsymbol{\beta}_{\boldsymbol{0}}$: intercept | -8.11538 | 0.02155 | -376.58 | p < 0.001 |
| $\boldsymbol{\beta}_{\boldsymbol{1}}$: time point | 0.07541 | 0.00424 | 17.78 | p < 0.001 |
| DM with cerebrovascular diseases admissions | | | | |
| $\boldsymbol{\beta}_{\boldsymbol{0}}$: intercept | -7.251687 | 0.009935 | -729.93 | p < 0.001 |
| $\boldsymbol{\beta}_{\boldsymbol{1}}$: time point | 0.084358 | 0.001941 | 43.47 | p < 0.001 |
| DM with cataract admissions | | | | |
| $\boldsymbol{\beta}_{\boldsymbol{0}}$: intercept | -7.604557 | 0.046891 | -162.175 | p < 0.001 |
| $\boldsymbol{\beta}_{\boldsymbol{1}}$: time point | 0.015244 | 0.009283 | 1.642 | 0.101 |
| DM with retinopathy admissions | | | | |
| $\boldsymbol{\beta}_{\boldsymbol{0}}$: intercept | -8.941096 | 0.028458 | -314.181 | p < 0.001 |
| $\boldsymbol{\beta}_{\boldsymbol{1}}$: time point | 0.049766 | 0.005599 | 8.888 | p < 0.001 |
| DM with amputation admissions | | | | |
| $\boldsymbol{\beta}_{\boldsymbol{0}}$: intercept | -8.955374 | 0.023377 | -383.09 | p < 0.001 |
| $\boldsymbol{\beta}_{\boldsymbol{1}}$: time point | 0.050946 | 0.004584 | 11.12 | p < 0.001 |

^1^ The negative binomial regression analysis with the number of admissions as the dependent variable and year as the explanatory variable were conducted to check the existence of temporal trend of admissions. The $\beta_{1}$ is the slope parameter of the variable that represents the year and the $\beta_{0}$ is the intercept.
Note: SE: Standard error, DM: Diabetes mellitus, CKD: Chronic kidney disease, MI: Myocardial infarction
